# Using machine learning-based radiomics to differentiate between glioma and solitary brain metastasis from lung cancer and its subtypes

**DOI:** 10.1007/s12672-023-00837-6

**Published:** 2023-12-06

**Authors:** Feng-Ying Zhu, Yu-Feng Sun, Xiao-Ping Yin, Yu Zhang, Li-Hong Xing, Ze-Peng Ma, Lin-Yan Xue, Jia-Ning Wang

**Affiliations:** 1 Department of Radiology, Affiliated Hospital of Hebei University, No.212 of Yuhua Road, Lianchi District, Baoding, 071000 China; 2 College of Electronic Information Engineering, Hebei University, Baoding, 071002 China; 3 College of Quality and Technical Supervision, Hebei University, No.180 of Wusi Road, Lianchi District, Baoding, 071002 China

**Keywords:** Glioma, Multiple layer perceptron, Non-small cell lung cancer, Radiomics, Small cell lung cancer

## Abstract

**Objective:**

To establish a machine learning-based radiomics model to differentiate between glioma and solitary brain metastasis from lung cancer and its subtypes, thereby achieving accurate preoperative classification.

**Materials and methods:**

A retrospective analysis was conducted on MRI T1WI-enhanced images of 105 patients with glioma and 172 patients with solitary brain metastasis from lung cancer, which were confirmed pathologically. The patients were divided into the training group and validation group in an 8:2 ratio for image segmentation, extraction, and filtering; multiple layer perceptron (MLP), support vector machine (SVM), random forest (RF), and logistic regression (LR) were used for modeling; fivefold cross-validation was used to train the model; the validation group was used to evaluate and assess the predictive performance of the model, ROC curve was used to calculate the accuracy, sensitivity, and specificity of the model, and the area under curve (AUC) was used to assess the predictive performance of the model.

**Results:**

The accuracy and AUC of the MLP differentiation model for high-grade glioma and solitary brain metastasis in the validation group was 0.992, 1.000, respectively, while the sensitivity and specificity were 1.000, 0.968, respectively. The accuracy and AUC for the MLP and SVM differentiation model for high-grade glioma and small cell lung cancer brain metastasis in the validation group was 0.966, 1.000, respectively, while the sensitivity and specificity were 1.000, 0.929, respectively. The accuracy and AUC for the MLP differentiation model for high-grade glioma and non-small cell lung cancer brain metastasis in the validation group was 0.982, 0.999, respectively, while the sensitivity and specificity were 0.958, 1.000, respectively.

**Conclusion:**

The application of machine learning-based radiomics has a certain clinical value in differentiating glioma from solitary brain metastasis from lung cancer and its subtypes. In the HGG/SBM and HGG/NSCLC SBM validation groups, the MLP model had the best diagnostic performance, while in the HGG/SCLC SBM validation group, the MLP and SVM models had the best diagnostic performance.

## Introduction

There is an increasing incidence of intracranial tumors with poor prognosis, especially in patients above 65 years of age. Gliomas approximately account for 30% of central nervous system (CNS) neoplasms, and 81% of CNS malignancies [1], wherein, the most common one is glioblastoma, with a five-year survival rate of 5% [2]. Brain metastasis is the most common type of tumor; common primary tumors include lung cancer, breast cancer, melanoma, and gastroenteric tumor, wherein, brain metastasis from lung cancer approximately accounts for 41–55% of all brain metastases [3]. Gliomas generally feature in a single lesion and occasionally in multiple lesions, while brain metastases feature in multiple lesions—only about 30% feature in a single lesion [4]. Previously, imaging differentiation mainly relied on conventional magnetic resonance imaging (MRI), and on some images, single brain metastasis and glioma could not be easily differentiated due to tumor enhancement, peri-tumor edema, and central necrosis, especially in patients with the initial symptom of single intracranial occupancy. Glioma and brain metastasis differ in origin. In the case of glioma, only the intracranial lesions are treated, while systemic conditions are rarely assessed. In the case of brain metastasis with unknown primary lesions, key sites should be screened to identify the primary lesions for developing the corresponding treatment plan. In addition to treatment, they also differ from each other in terms of staging, prognosis, and follow-up; therefore, it is important to carry out preoperative identification.

So far, some studies have conducted differential diagnoses with respect to high-grade glioma (HGG) and solitary brain metastasis (SBM) based on multiparametric MRI, and single and combined application of diffusion weighted imaging (DWI), perfusion weighted imaging (ASL, PWI), magnetic resonance spectroscopy (MRS), and diffusion tensor imaging (DTI), which have achieved some progress. Tsougos et al. [5] evaluated the role of 1HMRS, DWI, DTI, and DSCE in distinguishing glioblastoma from solitary brain metastases and found that peritumoral NAA/Cr, Cho/Cr, and rCBV were significantly distinguishable, with no significant difference between ADC and FA. Aslan et al. [6] found that the combination of ADCmin, rCBV, and Cho/NAA parameters in the peritumoral area was helpful in distinguishing SBM and HGG, with a diagnostic accuracy of 97%. Lin et al. [7] used 3D-ASL to differentiate between SBM and HGG and found that the CBF gradient of peritumoral edema may be more meaningful. Currently, no standards have been developed to apply the study findings into clinical practice, and limited by the scanning and post-processing conditions, the clinical applications of ASL, PWI, MRS, and DTI are not as widespread as conventional MRI.

With the development of radiomics, many scholars have combined it with conventional MRI for differential diagnosis of glioma and brain metastasis. Ortiz-Ramón et al. [8] analyzed T1WI of 50 brain metastases and 50 HGGs using six texture analysis methods and calculated 88 rotationally invariant texture features for each segmented lesion. The results showed that AUC reached 0.896, which indicated that the radiomics-based differentiation of 2D texture features extracted from MR images could accurately identify HGG and metastasis. Shrot et al. [9] applied machine learning to study 41 cases of glioblastoma, 38 cases of metastatic tumor, 50 cases of meningioma, and 12 cases of primary central nervous system lymphoma. They found that the accuracy, sensitivity, and specificity of binary SVM classification for glioblastoma, metastatic tumor, meningioma, and primary central nervous system lymphoma were 95.7%, 81.6%, and 91.2%, respectively; 92.7%, 95.1%, and 93.6%; 97%, 90.8%, and 58.3% and 91.5%, 90%, and 96.9%. Chen et al. [10] constructed thirty diagnostic models for distinguishing glioblastoma and brain metastasis, calculated the sensitivity, specificity, accuracy, and area under curve (AUC) of each model, and selected two models with an AUC of 0.80. There have been several related studies [11, 12], however, they mainly focus on the differentiation between HGG and SBM originating from a different primary focus. Approximately more than half of brain metastases are the metastasis from lung cancer, particularly SBM [3]. In clinical practice, larger SBMs originating from lung cancer and glioma are harder to differentiate [13, 14]. Therefore, we used the machine learning approach based on conventional T1WI-enhanced images in this study to explore the differentiation between gliomas and solitary brain metastases from lung cancer, and establishing a model, thus achieving preoperative accurate differentiation.

Due to the difference in the treatment of brain metastases from small cell lung cancer (SCLC) and non-small cell lung cancer (NSCLC), it is important to differentiate between them and gliomas. In this study, we also tried to establish a radiomics model based on machine learning, to differentiate between HGG and the subtypes of solitary brain metastasis from lung cancer.

## Materials and methods

### Study participants

We collected the data of 125 patients with gliomas that were pathologically confirmed at the Affiliated Hospital of Hebei University from January 2017 to September 2022. All these patients underwent preoperative contrast-enhanced T1WI (CE-T1WI), based on which, 20 patients with poor image quality were excluded. We collected the data of 800 patients with suspected brain metastases confirmed at the Affiliated Hospital of Hebei University from April 2016 to January 2022 based on the following inclusion criteria: primary lesions or metastases were pathologically confirmed; the primary lesion was lung cancer; the intracranial lesion was solitary; and the patients underwent CE-T1WI prior to the treatment of intracranial lesions. Finally, we included 105 patients with gliomas (70 males and 35 females, with the mean age of 52 and age range of 17–72) and 172 patients with SBM from lung cancer (103 males and 69 females, with the mean age of 62, and age range of 44–80). The patients with gliomas and those with brain metastasis from lung cancer were divided into the training group and validation group in an 8:2 ratio. This study was approved by the Ethics Committee of the Affiliated Hospital of Hebei University (No. HDFY-LL-2022-084). Due to the retrospective nature of this study, a waiver of informed consent was obtained.

### MRI scan

A 1.5 T MAGNETOM Amira MRI scanner and GE 3.0T Discovery MR750 scanner (Siemens) were used for scanning. CE-T1WI parameters were 1.5 T MAGNETOM Amira MRI (depth of stratum = 5 mm, TR = 339, TE = 3.54, matrix 256 × 256 mm); 3.0 T Discovery MR750 (depth of stratum = 5 mm, TR = 1750, TE = 24.5–25.2, matrix 256 × 256 mm); the contrast agent was gadopentetate dimeglumine (0.2 ml/kg).

### Image pre-processing

The original DICOM images were imported into ITK-SNAP (version: 3.8.0, http://itksnap.drg/) for manual outlining of the CE-T1WI images according to the following principles: (1) The outlining range was a high signal area on each CE-T1WI image, avoiding the edema, necrosis, and cystic areas; (2) If there was no high signal area, the low signal area would be filled with solid tumor, and all the area should be outlined as a low signal area. All images were outlined independently by two experienced CNS radiologists (with 5 and 7 years of experience), and they did not know the age, gender, clinical history, and pathological findings of the patients. If the difference in ROI between the two radiologists was smaller than 5%, the images could be fused directly, and if the difference in ROI was greater than 5%, a third senior radiologist (with 10 years of experience) was asked to determine the final outlining area.

### Feature extraction and modeling

#### Feature extraction

To improve the efficiency and extract high number of features from MRI images, we further processed the magnified ROI images with nine filters, including the origin (without a filter), square, square root, logarithm, gradient, wavelet transform, local binary pattern (LBP), and Laplacian of Gaussian (LoG). The first five filters converted based on image intensity and produced the corresponding derived images. It should be noted that wavelet transform can decompose each image into eight different frequency sub-bands. LBP is a simple and a computationally efficient method for obtaining gray level and rotationally invariant representation, which is a popular texture descriptor in medical image analysis. To enhance the edges of each image, LoG was performed for the original image. The original image was first smoothed and denoised by Gaussian kernel, then convoluted by the LoG kernel to highlight the areas with rapid intensity changes.

Several quantitative radiomics features were extracted to describe tumor heterogeneity. We extracted meaningful and reliable features from the NET area of the original image and the corresponding filtered images. A total of 1521 quantitative features were extracted from the images. They were subdivided into seven categories, including first-order statistics (304 features), shape (17 features), gray level co-occurrence matrix (GLCM, 384 features), gray level dependence matrix (GLDM, 224 features), gray level run matrix (GLRLM, 256 features), gray level size zone matrix (GLSZM, 256 features), and neighborhood graytone difference matrix (NGTDM, 80 features). Each category described tumor heterogeneity from its respective perspective. Therefore, these categories of features were deemed as multi-view features, which could provide additional information for the description of tumor heterogeneity.

#### Standardized processing and feature screening

To further improve the accuracy of classification, it is necessary to pre-process the extracted features, specifically, by normalizing and filtering them.

Due to the large differences between different features, Z-score method was used to normalize the features. To apply the Z-score method, it is necessary to calculate the mean and standard deviation of each feature vector. Then, the mean is subtracted from each feature and divided by the standard deviation, which helps improve the accuracy of classification. It can be calculated as follows:

1 $$z = \frac{{\left( {x - \mu } \right)}}{\sigma }$$

Due to the large number of features, we screened the features using Pearson’s Correlation Coefficient (PCC). The PCC was calculated for each pair of normalized features and if it was greater than the preset threshold, we removed it.

#### Data enhancement and modeling

**Fig. 1:**
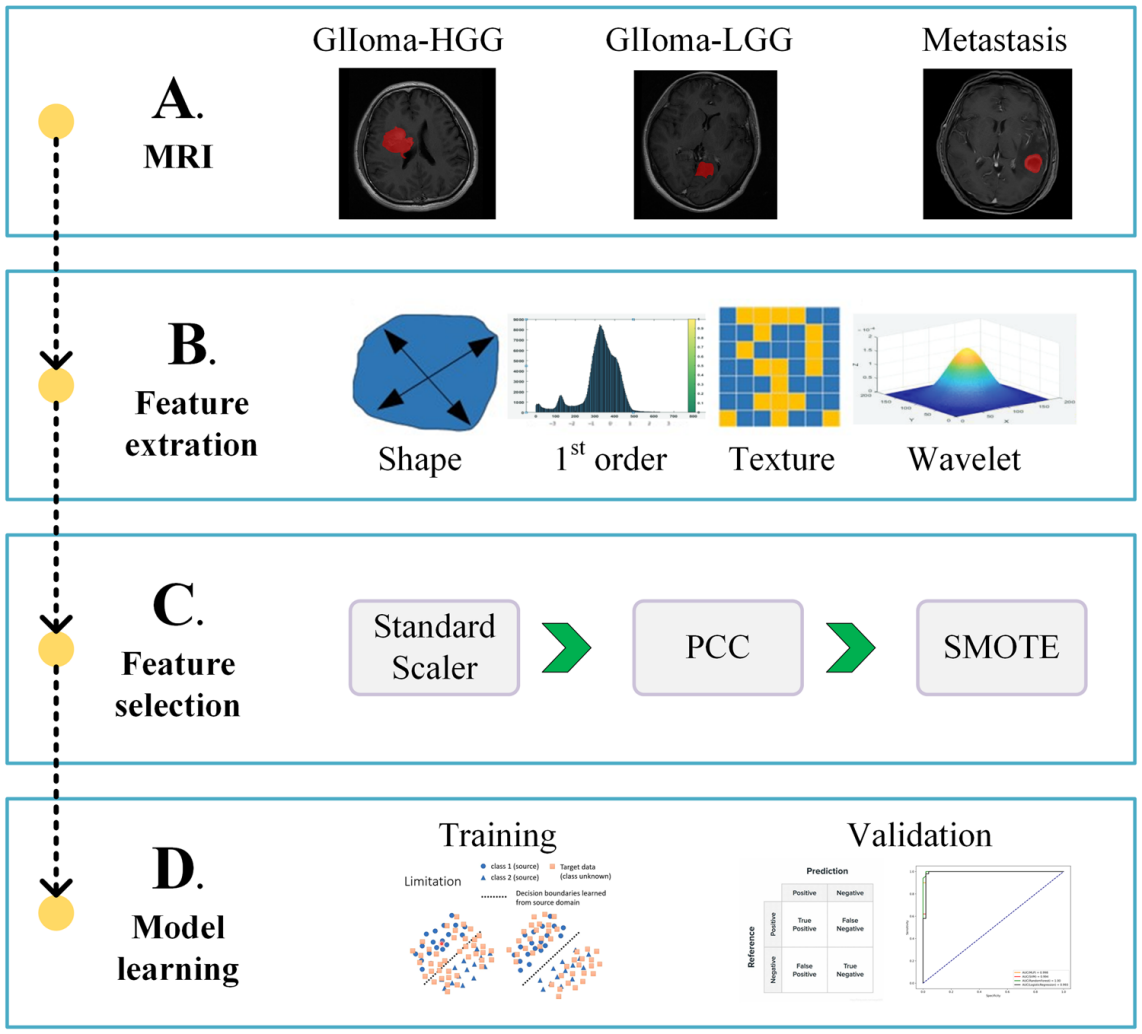
Workflow of the study

We established the authoritative multiple layer perceptron (MLP) classifier based on the PyTorch framework to explore the differentiation between glioma and solitary brain metastasis from lung cancer and its subtypes. The MLP classifier consists of three fully connected layers: at the first layer, the number of neurons was determined by the number of input features; while at the second and the third layers, there were 10 and 8 neurons, which were determined based on experience. To contrast with the MLP, we also adopted three authoritative classifiers, namely the support vector machine (SVM), random forest (RF), and logistic regression (LR). We calculated the sensitivity, specificity, accuracy, and area under curve (AUC) of the receiver operating characteristic (ROC) in the training group and validation group (Fig. 1).

## Results

### Features of patients

In this study, a total of 105 patients with gliomas were included, including 71 patients with HGG, and 34 patients with low-grade glioma (LGG). A total of 172 patients with lung cancer SBM were also included, with 34 patients with small cell lung cancer SBM, and 138 with non-small cell lung cancer (104 with lung adenocarcinoma, 24 with squamous carcinoma, 5 with adenosquamous carcinoma, 3 with large cell lung cancer, and 2 with other tumors). The patients were divided into the training group and validation group in an 8:2 ratio. There was no significant difference in gender and age between the groups (Table 1).

**Table1:** Patient characteristics table

	HGG	LGG	SCLC	NSCLC
Patients (277)	71	34	34	138
Age years (mean)	61	53	68	62
Male	48	22	20	83
Female	23	12	14	55

### Differentiation of high/low grade glioma and SBM

The data of high/low grade glioma were enhanced for 172 cases and the HGG/SBM and LGG/SBM differentiation models were established, and 12/14 features were extracted, respectively. The results showed that MLP classifier model in the validation groups had the best diagnostic efficiency; the accuracy of the HGG/SBM MLP classifier model in the validation group was 0.992 and AUC was 1.000; while the highest accuracy of the LGG/SBM MLP and RF classifier model in the validation group was 0.986 and the SVM classifier model with the highest AUC was 0.999. (The accuracy, sensitivity, specificity, and AUC of each classifier in the training and validation groups are shown in Tables 2 and 3.)

**Table 2:** Classification results for the HGG and SBM

	Val					Train				
	Accuracy	AUC	Precision	Sensitivity	Specificity	Accuracy	AUC	Precision	Sensitivity	Specificity
MLP	0.992	1.000	0.982	1.000	0.968	0.980	0.998	0.980	0.980	0.980
SVM	0.978	0.996	0.982	0.982	0.969	0.970	0.994	0.980	0.960	0.980
RF	0.989	1.000	0.983	1.000	0.969	0.950	1.000	0.909	1.000	0.900
LR	0.989	1.000	0.983	1.000	0.969	0.940	0.993	0.907	0.980	0.900

HGG, high-grade glioma; SBM, solitary brain metastasis; val, validation; MLP, multiple layer perceptron; SVM, support vector machine; RF, Random Forest; LR, Logistic regression

**Table 3:** Classification results for the LGG and SBM

	Val					Train				
	Accuracy	AUC	Precision	Sensitivity	Specificity	Accuracy	AUC	Precision	Sensitivity	Specificity
MLP	0.986	0.993	0.972	1.000	0.970	0.980	1.000	0.962	1.000	0.960
SVM	0.971	0.999	0.971	0.971	0.971	0.990	0.996	0.980	1.000	0.980
RF	0.986	0.995	1.000	0.971	1.000	0.971	1.000	0.971	0.971	0.971
LR	0.899	0.946	0.850	0.971	0.824	0.920	0.976	0.875	0.980	0.860

LGG, low-grade glioma; SBM, solitary brain metastasis; val, validation; MLP, multiple layer perceptron; SVM, support vector machine; RF, Random Forest; LR, Logistic regression

### Differentiation between HGG and brain metastasis from small cell lung cancer

The data of SBM from small cell lung cancer were enhanced for 71 cases, and the HGG/SCLC SBM differentiation models were established. Eleven features were extracted, and the results showed that the MLP and SVM classifier model in the validation groups had the best diagnostic efficiency; the accuracy of the MLP and SVM classifier model for HGG/SCLC SBM in the validation group was 0.966 and AUC was 1.000 (Table 4).

**Table 4:** Classification results for the HGG and SCLC SBM

	Val					Train				
	Accuracy	AUC	Precision	Sensitivity	Specificity	Accuracy	AUC	Precision	Sensitivity	Specificity
MLP	0.966	1.000	0.938	1.000	0.929	0.960	0.994	1.000	0.920	1.000
SVM	0.966	1.000	0.938	1.000	0.929	0.950	0.998	1.000	0.900	1.000
RF	0.931	0.995	0.933	0.933	0.929	0.910	0.996	1.000	0.820	1.000
LR	0.966	0.995	0.938	1.000	0.929	0.910	0.974	0.936	0.880	0.940

HGG, high-grade glioma; SCLC, small cell lung canser; SBM, solitary brain metastasis; val, validation; MLP, multiple layer perceptron; SVM, support vector machine; RF, Random Forest; LR, Logistic regression

### Differentiation between HGG and brain metastasis from non-small cell lung cancer

The data of HGG were enhanced for 138 cases, and the HGG/NSCLC SBM differentiation models were established. Thirteen features were extracted, and the results showed that the MLP classifier model in the validation groups had the best diagnostic efficiency; the accuracy of the MLP classifier model for HGG/SCLC SBM in the validation group was 0.982 and AUC was 0.999 (Table 5).

**Table 5:** Classification results for the HGG and NSCLC SBM

	Val					Train				
	Accuracy	AUC	Precision	Sensitivity	Specificity	Accuracy	AUC	Precision	Sensitivity	Specificity
MLP	0.982	0.999	1.000	0.958	1.000	0.970	0.993	0.961	0.980	0.960
SVM	0.964	0.996	0.985	0.985	0.969	0.960	0.994	0.942	0.980	0.940
RF	0.964	0.988	0.985	0.985	0.969	0.960	0.994	0.942	0.980	0.940
LR	0.929	0.992	0.885	0.958	0.906	0.940	0.978	0.940	0.940	0.940

HGG, high-grade glioma; SCLC, non-small cell lung canser; SBM, solitary brain metastasis; val, validation; MLP, multiple layer perceptron; SVM, support vector machine; RF, Random Forest; LR, Logistic regression

## Discussion

In this retrospective study, we differentiated between glioma and solitary brain metastasis from lung cancer and its subtypes by using a radiomics approach, and also adopted MLP, SVM, RF, and LR models. The results showed that the radiomics models had a high diagnostic efficiency in the differentiation of high grade glioma and solitary brain metastasis from lung cancer and its subtypes, in which, the MLP model had the best diagnostic efficiency in the validation groups.

One of the findings in this study was that the radiomics model was established for accurate preoperative differentiation of HGG and SBM. Since HGG had similar imaging findings to brain metastasis, especially solitary brain metastasis, the accuracy of preoperative diagnosis was low. Glioma and brain metastasis had different origins, as well as staging, screening, and treatment methods. Generally, gliomas only require focus on intracranial lesions, which rarely metastasize to other sites. As for brain metastasis with unknown primary lesions, key sites should be screened to identify the primary lesions for staging, to determine the subsequent treatment. Therefore, preoperative differentiation played an important role. Although the operation or aspiration biopsy can differentiate between the two tumors, when the tumor was close to or in a functional area of the brain or when the patient was too weak to undergo the operation, only the non-invasive mode could be selected for differentiation [15]. Conventional MRI is the most commonly used imaging differentiation method, however, it has low accuracy, for example, HGG was the most common type with peritumor enhancement of T1WI (about 40%), followed by tumor metastasis (about 30%) [16]. Some studies showed that multi-parameter MRI could better differentiate between the two tumors [5–7]. In the case of multiparametric joint application, Kerim et al. [6] established an optimal model for the differentiation of HGG and brain metastasis, with an AUC of 0.970. However, the results are affected by the scanning machine, scanning conditions, and the post-processing methods used in clinical practice [17].

The radiomics approach has become a promising method for tumor diagnosis, outcome prediction, and prognosis evaluation [18]. It was found to have outstanding advantages in the analysis of different types of tumors and their prognosis. Currently, studies have been conducted involving the lung, liver, cardiovascular, and neurological areas [19–22]. Moran et al. [23] analyzed 212 patients with glioblastoma and 227 patients with brain metastasis based on machine learning, and concluded that the SVM classifier could best differentiate between the glioblastoma and brain metastasis, with the average accuracy of 0.85, sensitivity of 0.86, specificity of 0.85, and AUC of 0.96. In this study, the accuracy and AUC of the four HGG/SBM classifiers were higher than those in the previous studies; MLP had the highest comprehensive diagnostic effectiveness, and the accuracy and AUC in the validation groups of the HGG/SBM were 0.992 (accuracy) and 1.000 (AUC), respectively. Karoline et al. [24] demonstrated that the heterogeneity of the solid part of the tumor had no significant difference; however, the heterogeneity of GBM peritumoral edema was significantly higher than that of solitary brain metastasis, with the sensitivity and specificity of 80% and 90%, respectively. In contrast to the study conducted by Karoline et al., Qin et al. [25] extracted the features of the solid part of the tumor, differentiated between glioblastoma and SBM based on machine learning, and obtained an AUC of 0.94 and accuracy of 95%. Our results are consistent with the results of their study. Machiko et al. [26] found that machine learning based on a combination of texture parameters from multiple sequences outperformed machine learning from individual sequences. The AUC of the model for differentiating HGG and brain metastasis in combination with T2WI, CE-T1WI, and ADC was 0.92, and the AUC of a simple sequence was inferior to the results of the combination. Studies have used radiomics methods based on CT, PET, and PET-CT fusion to predict the survival of patients with head and neck squamous cell carcinoma, and have achieved good results [27, 28]. Here, we studied the single sequence of CE-T1WI The reasons are as follows: (1) In the delineation and feature extraction of ROI, there are more single sequences with less workload and shorter time required. This study attempts to explore simpler, faster, and more efficient radiomics methods for distinguishing; (2) Some lung cancer patients only underwent a single sequence examination to determine whether there was intracranial metastasis. In order to obtain more data and make the model more accurate, this study first only used CE-T1WI images. We obtained better results. As for the reasons, firstly, the authoritative algorithm SVM was generally used in the past, however in this study, we used MLP, which was confirmed to be significantly superior to SVM. Secondly, previous studies focused on the differentiation of HGG and brain metastases of multiple primary foci (lung, breast, gastrointestinal tract, etc.); whereas in clinical practice, the larger SBM from lung cancer was harder to be differentiated from glioma, and there was a higher incidence of brain metastasis from lung cancer. Therefore, the results of this study are more accurate and have greater clinical significance.

As per the results of several studies, the most commonly used classifiers for differentiating glioma and tumor metastasis were support vector machine (SVM) and k-nearest neighbor (KNN); SVM performed well, and as a traditional machine learning method, it is a commonly used algorithm in neuroradiological predictive modeling by virtue of its simplicity and flexibility [29]. We used the MLP classifier in this study. With a wide application scope and strong scalability, MLP can fit complex functions with a generic function approximation method, and solve the non-linear classification problem. As no classifier is universally considered the best and accepted in clinical practice, we compared MLP and the three authoritative classifiers, and evaluated their diagnostic efficiency in this study. The results revealed that MLP had good diagnostic efficiency in the validation group, and the results of other classifiers were higher than the previous diagnostic efficiency. New directions and ideas have been provided for future radiomics research to distinguish between gliomas, metastatic tumors, and other brain tumors. Our research also utilized data augmentation methods to address the most common issue of small data volume in radiomics training models.

In this study, we explored the machine learning method for preoperative differentiation of low-grade glioma and brain metastasis. For most low-grade gliomas, especially those that are Grade I as classified by the WHO guidelines, the MRI showed a low signal without significant enhancement, and no edema, necrosis, or cystic areas. The imaging findings of these gliomas and brain metastases were significantly different, and they could be easily differentiated preoperatively. Some lesions tent to have similar imaging findings to brain metastases, such as ring enhancement and peritumoral edema. Peritumoral edema of low-grade glioma and brain metastasis may be caused by secondary ischemia due to the stress of the tumor on the peripheral cerebral parenchyma; therefore, they cannot be easily differentiated based on conventional MRI and even functional MRI (such as MRS and ASL). In this study, a total of 14 features extracted by machine learning were used for modeling and differentiating LGG/SBM, and a higher accuracy and AUC were obtained.

Another finding in this study is that the radiomics model based on machine learning can differentiate between HGG and brain metastasis from small cell lung cancer/non-small cell lung cancer. At present, there is no research on distinguishing subtypes of gliomas from solitary lung cancer brain metastases and lung cancer brain metastases. Small cell lung cancer and non-small cell lung cancer have different treatment modalities, and the treatment of brain metastasis may differ greatly. As for patients with asymptomatic brain metastasis from NSCLC, systemic therapy can be performed, and as for symptomatic patients with no more than three brain metastases, surgery, stereotactic radiotherapy (SRT), or SRT combined with whole brain radiotherapy (WBRT) can be performed. For patients with asymptomatic brain metastasis from SCLC, systemic chemotherapy can be performed, followed by WBRT, while for symptomatic patients, WBRT can be performed actively [30, 31]. Patients with brain metastasis from SCLC and NSCLC are treated with different chemotherapy drugs [32, 33]. In this study, we explored a method that can differentiate between brain metastasis from SCLC/NSCLC and glioma, and its diagnostic efficiency was high. Therefore, it can identify whether the primary focus in the lung is small cell lung cancer, thereby providing a reference for clinical treatment and medication; furthermore, some patients cannot undergo surgery or aspiration biopsy to determine the pathological types of lung cancer due to physical or other reasons.

This study has several limitations, firstly, as a retrospective study, the sample may be biased; secondly, the data volume of high/low grade glioma was small—there were only 34 patients with low grade glioma, and 172 patients with glioma, showing a big difference in data, however, the data enhancement method adopted in this study reduced the difference. ROI was outlined manually, showing strong subjectivity, great workload, and low efficiency. The data in the training and validation groups were collected from the same hospital, and were not validated by an external validation set; they had the same scanning parameters and a single source of patients. Another drawback of this study is that the clinical symptoms and laboratory indicators of gliomas and brain metastases were not included in the study. We only used machine learning methods and did not explore deep learning methods. In terms of experimental design, only a single sequence CE-TIWI was used to study the tumor enhancement area, without including edema around gliomas and metastatic tumors, adjacent cortical signal changes, other sequences other than CE-T1WI, or CT, PET, and other images. When delineating ROI, there is also uncertainty in determining the boundary of the necrotic zone. In the future, these shortcomings can be resolved by expanding the sample size, working with multiple centers, including the clinical symptoms and laboratory indicators of gliomas and brain metastases, and exploring automatic outlining of ROI [34–36].

To summarize, the MRI radiomics model has a certain application value for the differentiation of glioma and solitary brain metastasis from lung cancer and its subtypes, and can provide more information for clinical evaluation, staging, medication, and prognosis, thereby offering new ideas for precise differentiation of glioma and solitary brain metastasis from lung cancer and its subtypes. In the future, we can also conduct research on machine learning or deep learning based on tumor surrounding edema, adjacent cortical signal changes, sequences other than CE-T1WI, or images such as CT and PET.

## Data Availability

All data generated or analysed during this study are included in this article. Further enquiries can be directed to the corresponding author.
